# PERSONS LIVING WITH COGNITIVE IMPAIRMENT SHARE THEIR VIEWS ON TECHNOLOGY

**DOI:** 10.1093/geroni/igz038.3459

**Published:** 2019-11-08

**Authors:** Susan Wehry, Regula H Robnett

**Affiliations:** 1 University of New England College of Osteopathic Medicine, Biddeford, Maine, United States; 2 University of New England, Portland, Maine, United States

## Abstract

The purpose of this study was to examine the experience of adults living with cognitive impairments and that of their care partners with digital technology including current use of, ease with and openness to using smart assistive technologies (SATs). SATs for older adults with (and without) cognitive impairments have become increasingly commonplace. Research on various digital devices has focused primarily on supporting users’ independence and care partner concerns for safety and security. Our qualitative, interview-based research project provided digital devices chosen by participants to address a specific personal goal. Interviews were conducted in the home and set-up assistance was provided during the initial interview. At the conclusion of the trial period, a second interview was conducted in the home. We describe the participants’ commendations for, expectations of, and frustrations with current technology as well as recommendations for potential, helpful digital technology. Current technology offers great promise but a disconnect between the design of digital technologies and the needs and wishes of the end-user still exists. This study will help inform additional user-driven application SATs, including those aimed at enhancing enjoyment and a higher quality of life.

